# Plasma lipidomic profiles of kidney, breast and prostate cancer patients differ from healthy controls

**DOI:** 10.1038/s41598-021-99586-1

**Published:** 2021-10-13

**Authors:** Denise Wolrab, Robert Jirásko, Ondřej Peterka, Jakub Idkowiak, Michaela Chocholoušková, Zuzana Vaňková, Karel Hořejší, Ivana Brabcová, David Vrána, Hana Študentová, Bohuslav Melichar, Michal Holčapek

**Affiliations:** 1 Department of Analytical Chemistry, Faculty of Chemical Technology, University of Pardubice, Studentská 573, 532 10 Pardubice, Czech Republic; 2 Department of Oncology, Faculty of Medicine and Dentistry, Palacký University and University Hospital, I.P. Pavlova 6, 775 20 Olomouc, Czech Republic; 3 Present Address: Comprehensive Cancer Center Nový Jičín, Hospital Nový Jičín, Nový Jičín, Czech Republic

**Keywords:** Cancer screening, Tumour biomarkers, Biomarkers, Analytical chemistry

## Abstract

Early detection of cancer is one of the unmet needs in clinical medicine. Peripheral blood analysis is a preferred method for efficient population screening, because blood collection is well embedded in clinical practice and minimally invasive for patients. Lipids are important biomolecules, and variations in lipid concentrations can reflect pathological disorders. Lipidomic profiling of human plasma by the coupling of ultrahigh-performance supercritical fluid chromatography and mass spectrometry is investigated with the aim to distinguish patients with breast, kidney, and prostate cancers from healthy controls. The mean sensitivity, specificity, and accuracy of the lipid profiling approach were 85%, 95%, and 92% for kidney cancer; 91%, 97%, and 94% for breast cancer; and 87%, 95%, and 92% for prostate cancer. No association of statistical models with tumor stage is observed. The statistically most significant lipid species for the differentiation of cancer types studied are CE 16:0, Cer 42:1, LPC 18:2, PC 36:2, PC 36:3, SM 32:1, and SM 41:1 These seven lipids represent a potential biomarker panel for kidney, breast, and prostate cancer screening, but a further verification step in a prospective study has to be performed to verify clinical utility.

## Introduction

Cancer incidence and mortality are increasing worldwide as a result of population aging and changing patterns of other risk factors^1^. Malignant disorders are categorized according to the site where tumor growth has started, regardless of subsequent metastatic spread to other parts of the body^2^. Prostate cancer is the second most frequently diagnosed cancer in men^3^. Breast cancer is the most frequently diagnosed cancer and one of the main causes of cancer-related death in women^4^. On the other hand, kidney cancer is the 9th most common cancer in men and the 14th most common cancer in females^5^. However, there is a marked geographical variation in the incidence rate with the highest incidence rates of kidney cancer for men in the Czech Republic among all European countries^6^. The first tests to detect prostate cancer include the levels of prostate specific antigen (PSA) in peripheral blood and the digital rectal examination (DRE). In case of abnormal DRE or elevated PSA levels, a transrectal ultrasound-guided biopsy is performed for verification^7^. Mammography represents the principal method for detecting breast cancer screening, but the diagnosis is commonly supported by other imaging methods including magnetic resonance imaging, positron emission tomography, computed tomography, or single‐photon emission computed tomography^8^. Kidney cancer is often discovered by chance during examination with imaging methods for another purpose^9^, such as ultrasound, computed tomography, or magnetic resonance imaging^10^.

Generally, all imaging methods are subject to the limitation that very small tumors are not properly visualized, resulting in low sensitivity for early stage^11^. Staging examinations are performed after cancer diagnosis based on information regarding the location, spread, and extent of the tumor^12^. The diagnosis and treatment of patients in the early stage of cancer increase the chance for survival and cure compared to patients diagnosed at the late stage.

Cancer screening methods aim at the early detection of cancer for high-risk individuals^13^. Nowadays, more attention is devoted to the development of cancer screening methods based on the examination of peripheral blood, including liquid biopsy. The analysis of circulating cells, platelets, extracellular vesicles, mRNA, miRNA, proteins, cell-free DNA (cfDNA), and circulating tumor DNA (ctDNA) in blood are investigated as potential approaches in cancer screening^14^. Recently, the successful detection of eight cancer types based on the analysis of proteins and mutations in ctDNA in blood was reported^15^. Metabolomics also attracts research attention in cancer screening^16^ and other metabolic disorders^17^. Lipidomics can be considered as a part of metabolomics that deals with the comprehensive analysis of lipids, which are important biomolecules involved in many biological processes, such as signaling molecules or constituents of cell membranes, energy storage, and various other metabolic pathways^18^. Clinical lipidomics revealed that plasma lipid concentrations can be changed for various malignant diseases^19,20^.

Here, our aim is to quantitatively determine the plasma lipidome of patients with kidney, breast, and prostate cancer and compare it with the lipidome of healthy controls. Lipidomic changes are investigated and visualized using various multivariate data analysis (MDA) tools, such as principal component analysis (PCA), orthogonal projection to latent structures discriminant analysis (OPLS-DA), and receiver operating characteristics (ROC). The ultrahigh-performance supercritical fluid chromatography—mass spectrometry (UHPSFC/MS) is used as a powerful high-throughput and sensitive method for quantitative lipidomic analysis based on the lipid class separation approach recommended for reliable quantitation together with the use of exogenous internal standards for individual lipid classes^21,22^.

## Results

### Study design

Heparin plasma samples from 289 cancer patients and 192 volunteers without the history of previous malignant disease (further referred to as healthy controls) were obtained. Patients were diagnosed with breast, prostate, or kidney cancer based on standard medical procedures at the University Hospital in Olomouc.

**Figure 1:**
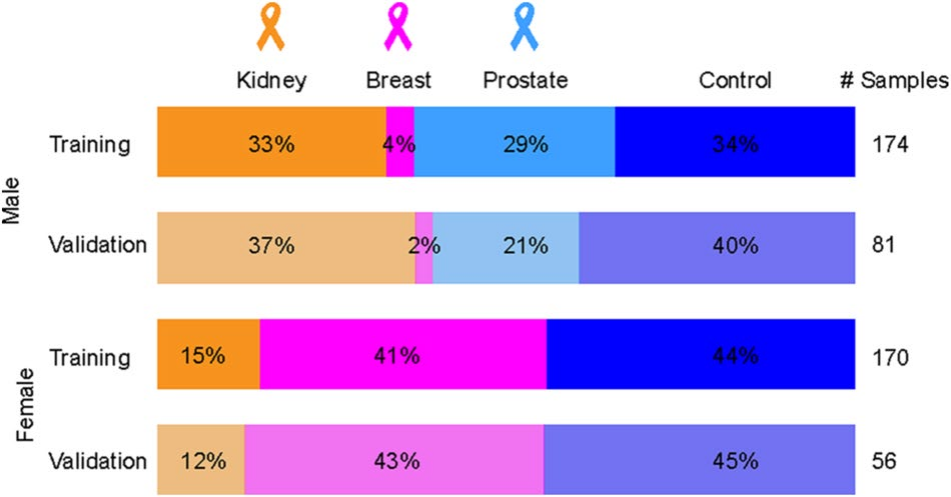
Overview of the sample set (n = 481) used for the study of 3 cancer types. Samples were divided into training (75%) and validation sets (25%). Plasma samples obtained from patients with kidney (n = 119), breast (n = 103) and prostate (n = 67) cancers and healthy controls (n = 192) were included in the study.

The sample set was divided into training and validation sets, whereby about 25% of samples for each cancer type and healthy controls were assigned to the validation set. Finally, the training set included 135 healthy controls, 209 cancer patients (77 breast, 82 kidney, and 50 prostate cancers), and the validation set included 57 healthy controls and 80 cancer patients (26 breast, 37 kidney, and 17 prostate cancers). The overview of all samples together with the clinical information is summarized in Fig. 1 and Supplementary Tables S1 and S2. The average age of healthy volunteers was lower than that of cancer patients, and the average body mass index (BMI) was comparable for both sample groups. Cancer patients are classified according to the TNM system. The majority of samples are assigned as T1 stage, typically for breast cancer (59%) and kidney cancer (47%), while T2 stage is predominant for prostate cancer (69%).

Previous studies reported differences in plasma lipidome depending on gender^23–27^. As a consequence, the gender effect on the prediction performance using MDA for both genders and gender-separated models was evaluated (Supplementary Fig. S1) using OPLS-DA models. The accuracy was slightly higher for gender-separated models, in particular, for females. Therefore, the sample set was divided according to gender. Obviously, prostate cancer occurs only in men and the overwhelming majority of breast cancer patients are women, so the gender separation is an important issue only for kidney cancer, where this study has 73% of men and 27% of women samples.

### Discovery phase measured by UHPSFC/MS

The order of samples was randomized separately for the extraction and UHPSFC/MS measurements to exclude any possible biases. The lipidomic analysis of human plasma in the discovery phase resulted in the quantitation of 138 lipids (Supplementary Table S3a) belonging to glycerolipids, glycerophospholipids, and sphingolipids.

**Figure 2:**
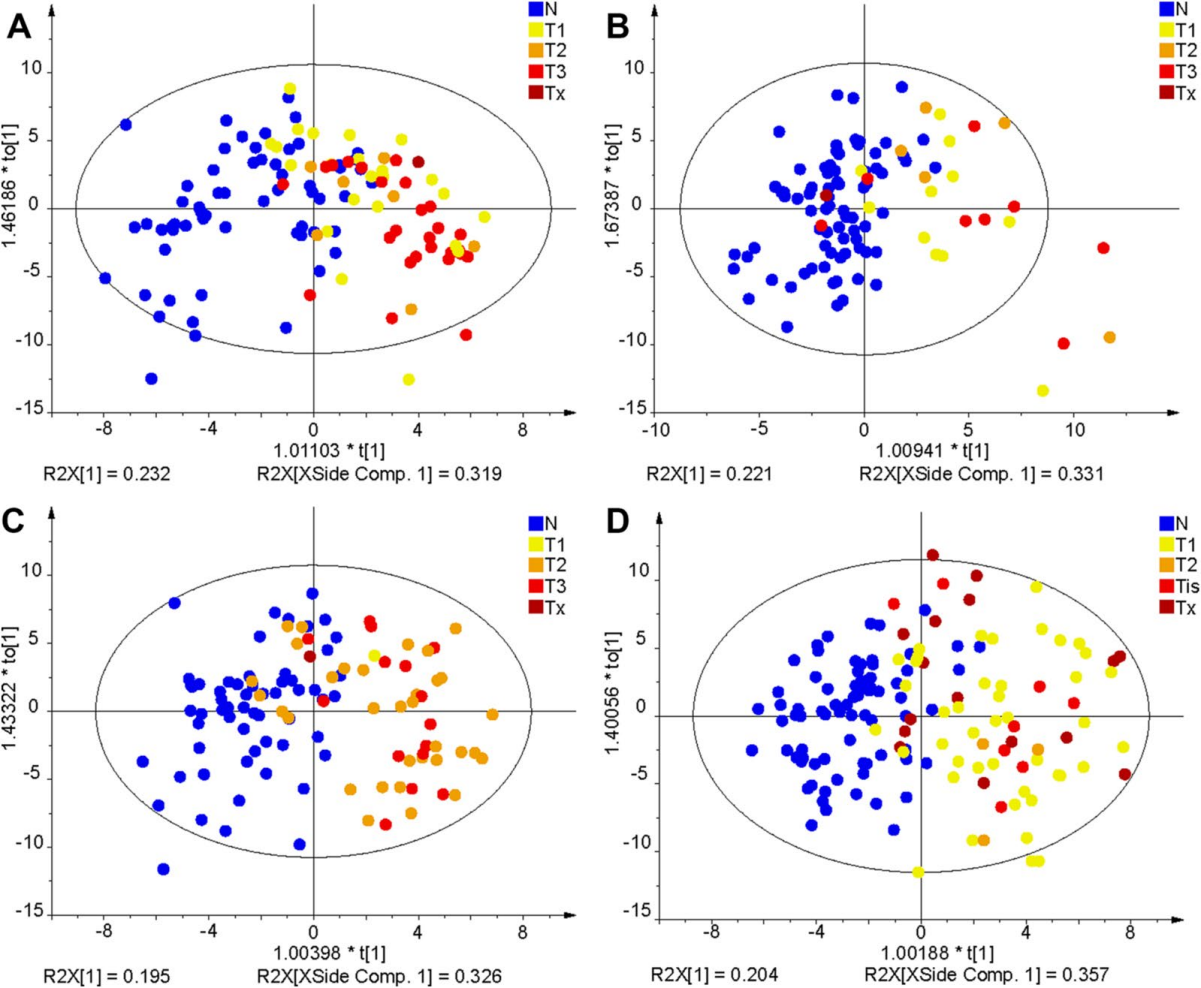
OPLS-DA models used for the differentiation of cancer and control plasma samples. The training set from the discovery phase was measured by UHPSFC/MS and then used to build OPLS-DA models: (**A**) kidney cancer versus control samples for males, (**B**) kidney cancer versus control samples for females, (**C**) prostate cancer versus control samples for males, and (**D**) breast cancer versus control samples for females. Annotation: blue—healthy controls (N), yellow—cancer stage T1 (T1), orange—cancer stage T2 (T2), light red—cancer stage T3 or Tis (T3/Tis), and dark red—unknown cancer stage (Tx).

**Figure 3:**
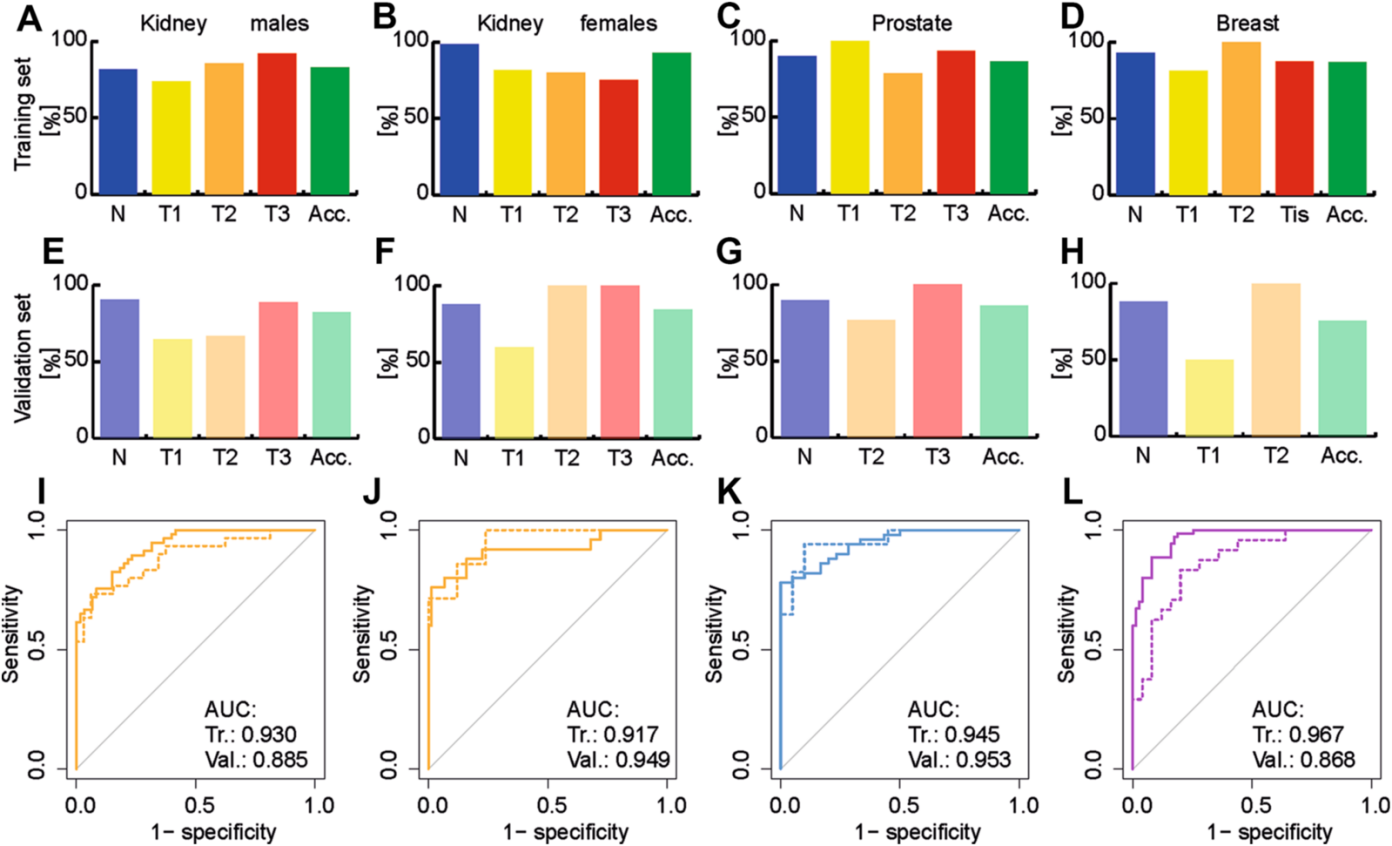
OPLS-DA models were used to predict the pathological state of human subjects. The training set was used to build OPLS-DA models. The percentage of specificity (blue), sensitivity (yellow, orange, red), and accuracy (green) for training and validation sets using UHPSFC/MS data from the discovery phase are presented. The sensitivity was determined for each stage of cancer (yellow—T1, orange—T2, and red—T3), excluding samples with unknown cancer stage. Training set: (**A**) kidney cancer versus healthy control for males, (**B**) kidney cancer versus healthy control for females, (**C**) prostate cancer versus healthy control for males, and (**D**) breast cancer versus healthy control for females. Validation set: (**E**) kidney cancer versus healthy control for males, (**F**) kidney cancer versus healthy control for females, (**G**) prostate cancer versus healthy control for males, and (**H**) breast cancer versus healthy control for females. ROC curves with the corresponding AUC values are presented, where the continuous lines represent the ROC curve for the training set, and dashed lines for the validation set. (**I**) kidney cancer versus healthy control for males, (**J**) kidney cancer versus healthy control for females, (**K**) prostate cancer versus healthy control for males, and (**L**) breast cancer versus healthy control for females.

Non-supervised PCA and supervised OPLS-DA were applied for all training set samples to visualize differences between sample groups (healthy controls and cancer patients) for the three cancer types studied (Fig. 2A–D). MDA allows the prediction of samples to belong to a particular sample group. The samples of the validation set were predicted by the corresponding OPLS-DA model built on the samples of the training set samples (Supplementary Tables S4 and S5). The specificity, sensitivity, and accuracy values for individual models were the following: kidney cancer males—91%, 73%, and 82%; kidney cancer females—88%, 71%, and 84%; breast cancer females—88%, 63%, and 76%; prostate cancer—90%, 82%, and 87%. Specificity and sensitivity values depending on the cancer stage and accuracy values depending on the cancer type are summarized in Fig. 3 for training and validation sets. The prediction performance is only slightly higher for the training set than for the validation set, which is an important confirmation that the statistical models do not collapse for the prediction of samples with unknown classification. ROC curves for the diagnostic ability to classify healthy control and cancer patient samples are illustrated in Fig. 3I–L for individual cancer types. The AUC values for the different types of cancer types ranged from 0.917 to 0.967 for the training set and from 0.868 to 0.953 for the validation set.

### Qualification phase measured by UHPSFC/MS

**Figure 4:**
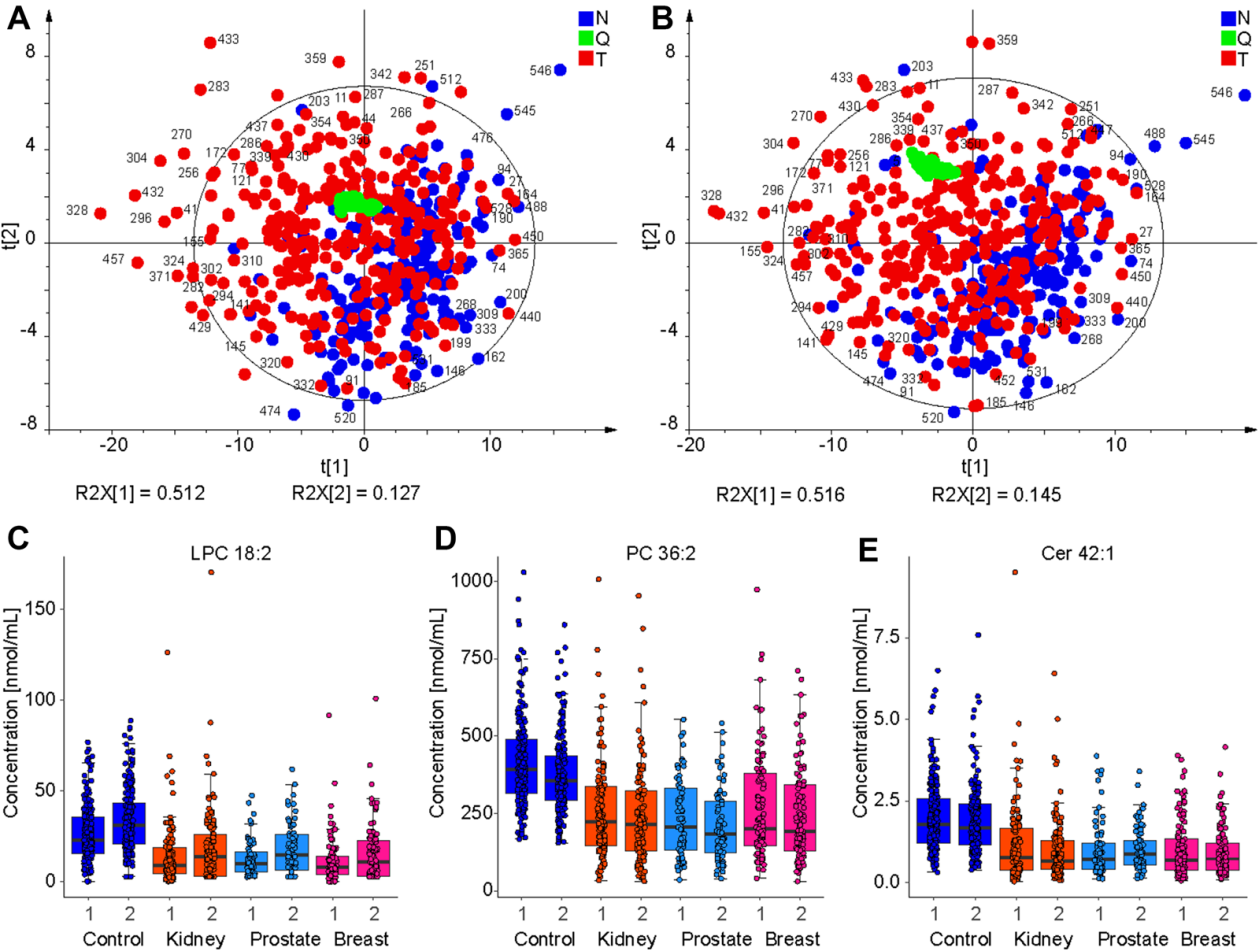
Comparison of UHPSFC/MS results in discovery and qualification phases. (**A**) PCA of all samples (validation and training sets) in the discovery phase. (**B**) PCA of all samples (validation and training sets) in the qualification phase (red—cancer (T), blue—control (N), and green—QC). Selected samples were annotated for comparison purposes. Boxplots comparing the concentrations of samples obtained from patients with different pathological states (blue: control, orange: kidney cancer, light blue: prostate cancer, and pink: breast cancer) for the discovery phase (1) and qualification phase (2) for (**C**) LPC 18:2, (**D**) PC 36:2, (**E**) Cer 42:1 using UHPSFC/MS.

**Figure 5:**
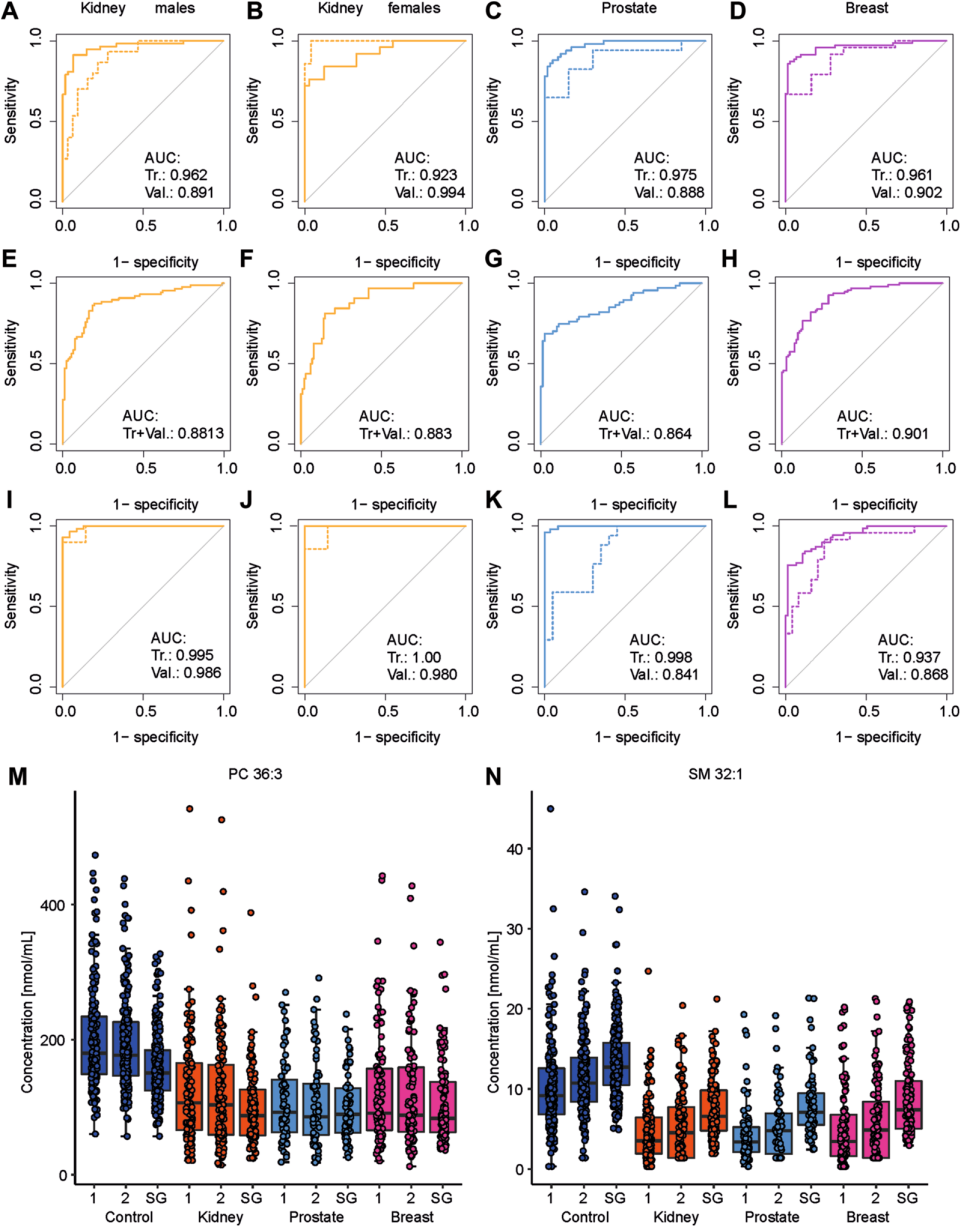
ROC curves with the corresponding AUC values are presented, where the continuous lines represent the ROC curve for the training set and dashed lines for the validation set using UHPSFC/MS. (**A**) kidney cancer versus healthy control for males, (**B**) kidney cancer versus healthy control for females, (**C**) prostate cancer versus healthy control for males, and (**D**) breast cancer versus healthy control for females using data from the qualification phase for MDA, and (**E**) kidney cancer versus healthy control for males, (**F**) kidney cancer versus healthy control for females, (**G**) prostate cancer versus healthy control for males, and (**H**) breast cancer versus healthy control for females predicting the data from the qualification phase using the discovery phase for MDA, (**I**) kidney cancer versus healthy control for males, (**J**) kidney cancer versus healthy control for females, (**K**) prostate cancer versus healthy control for males, and (**L**) breast cancer versus healthy control for females using shotgun MS data for MDA. Box plots comparing concentrations of samples obtained from patients with different pathological states (blue: healthy control, orange: kidney cancer, light blue: prostate cancer, and pink: breast cancer) for discovery (1) and qualification (2) phases using UHPSFC/MS and shotgun MS (SG): (**M**) PC 36:3, and (**N**) SM 32:1.

UHPSFC/MS measurements were repeated after several months to verify the repeatability of results, and these repeated measurements are called as the qualification phase. The same sample extracts were measured using different sample measurement sequences to minimize the risk of hidden biases. In total, 138 lipids were also quantified in the qualification phase (Supplementary Table S6a), with 126 of 138 lipids (91%) quantified in both the discovery and qualification phase. The small difference is caused mainly by low abundant short fatty acyl glycerolipids close to the limit of quantitation. PCA score plots for UHPSFC/MS measurements were compared in Fig. 4A,B. Both data sets show the quality control (QC) sample (green) cluster in the PCA score plot, indicating satisfactory method stability during the measurement sequence. The partial group separation between cancer (red) and control (blue) groups is already observed in non-supervised PCA score plots, which confirms a high reproducibility of the lipidomic profiling, as illustrated by numbers of selected samples in Fig. 4A,B. The first and second data sets were compared by calculating the relative standard deviation (RSD) for each lipid in each sample (Supplementary Table S7). In total, 65% of all values have RSD < 20%, and the average of all RSD for each lipid and all samples is 19%. Figure 4C–E further illustrates the reproducibility of quantitative results for selected dysregulated lipids in the discovery and qualification phases, as the medians of box plots for the first and second measurements are comparable. Furthermore, the box plots also show that the selected lipid species are downregulated in all cancer types compared to the control group (Fig. 4C–E). MDA was also applied for repeated measurements using the training sample set for building models. Generally, the prediction performance was comparable for both phases (summary in Supplementary Tables S4 and S5). ROC curves are shown in Fig. 5A–D for all cancer types. AUC values ranged from 0.888 to 0.994. Furthermore, MDA models for the discovery phase were used to predict the sample set of the qualification phase (Supplementary Tables S4 and S8). Specificity, sensitivity, and accuracy values for individual models were the following: kidney cancer males—62%, 91%, and 76%; kidney cancer males—86%, 72%, and 83%; breast cancer females—94%, 57%, and 76%; prostate cancer—88%, 75%, and 82%. ROC curves are summarized for all samples in Fig. 5E–H for all types of cancer. AUC values ranged from 0.864 to 0.901.

### Shotgun MS measurements

To further verify the conclusions of UHPSFC/MS measurements, all samples were also measured by shotgun MS as an independent alternative technique. 412 lipid species were quantified by shotgun MS for the categories of glycerolipid, glycerophospholipid, and sphingolipid (Supplementary Table S6c), which is about 3 times more quantified lipid species compared to UHPSFC/MS. The MDA prediction performance was comparable (Supplementary Tables S4 and S5). ROC curves for training and validation sets are summarized in Fig. 5I–L for all types of cancer. AUC values ranged from 0.841 to 1.00. For two of the most significantly regulated lipid species, the box plots are presented in Fig. 5M,N for all pathological states and data sets for both phases of UHPSFC/MS (1, 2) and shotgun MS (SG).

### Influence of the number of lipid species on the prediction capability of statistical models

**Figure 6:**
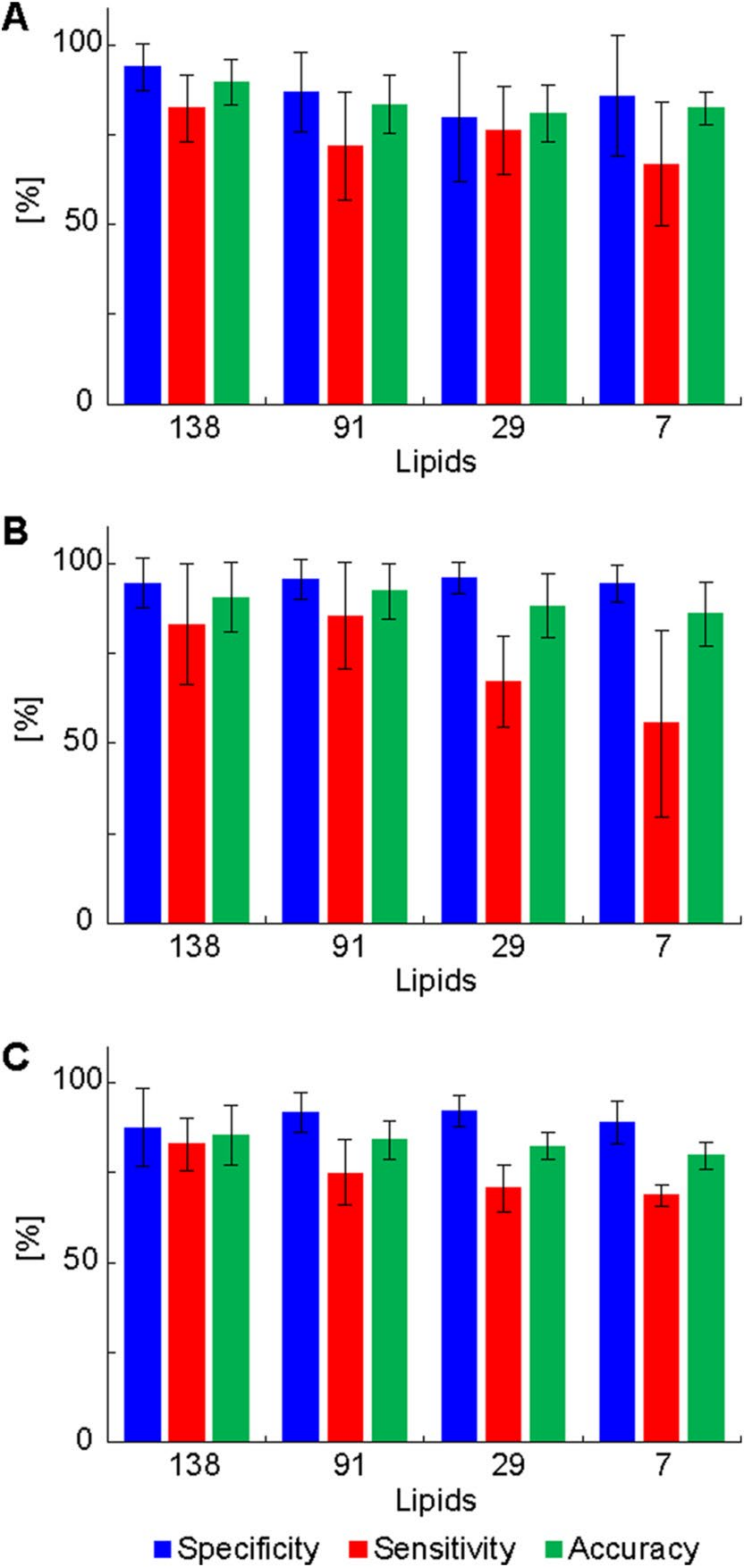
Influence of decreased number of lipid species used for MDA using UHPSFC/MS data in the discovery phase. (138 lipids: no exclusion, 91 lipids: common lipids from the discovery and qualification phase using UHPSFC/MS and shotgun MS, 29: only lipid species included, which are significant according to the Bonferroni correction for at least 10 from 15 models. SM 38:1 and SM 42:1 were excluded, because these variables were only significant for UHPSFC/MS data sets (10/10) and therefore a method bias cannot be excluded. 7: only lipid species included, which are significant according to the Bonferroni correction for at least 14 from 15 models. The average of the specificity (blue), sensitivity (red), and accuracy (green) for validation and training set as well as for both genders were calculated. (**A**) kidney cancer, (**B**) breast cancer, and (**C**) prostate cancer.

The influence of the number of quantified lipid species used for MDA on the accuracy to correctly classify samples was investigated. First, only lipid species common for both phases with UHPSFC/MS and shotgun MS were selected. In total, 91 common lipid species were used for MDA in each data set. The overall prediction performance was comparable to that obtained when all quantified lipid species were used for MDA, independent of the method and diagnosis (Supplementary Tables S4 and S9). The number of lipid species in MDA models was further reduced by considering additional statistical criteria, such as fold change (more than ± 20%), *p*-value (< 0.05), and VIP value (> 1). Supplementary Table S10 provide information how variables were reduced. The whole data set was divided into 5 data subsets (healthy control vs. kidney cancer samples for males and females, healthy control vs. breast cancer samples for males and females, and healthy control vs. prostate cancer samples for males) for UHPSFC/MS (1st and 2nd measurements) and shotgun MS, which resulted in 15 data subsets. In total, 29 lipid species were statistically significant after the Bonferroni correction for at least 10 from 15 data subsets considering all cancer types, methods, and measurements. The accuracy slightly decreased with decreasing number of lipids used for MDA independent of the investigated cancer type and method (Supplementary Tables S4 and S11). However, as the decrease of the prediction performance was not so pronounced, the effect of further reduction of lipids used for MDA on the prediction performance to correctly assign the sample type was investigated. CE 16:0, Cer 42:1, LPC 18:2, PC 36:2, PC 36:3, SM 32:1, and SM 41:1 were significant according to the Bonferroni correction for at least 14 of the 15 data subsets considering all cancer types, methods and measurements. MDA was performed for these 7 lipid species and the prediction performance was evaluated (Supplementary Tables S4 and S12). The average of sensitivity, specificity, and accuracy values for the different number of lipids considering all methods and genders was calculated (Fig. 6). Generally, the sensitivity and consequently the accuracy decreased with decreasing number of lipids used for MDA. The specificity was not affected by the number of lipid species, independent of the cancer type (Fig. 6). No effect of the cancer stage on concentrations of the most significant lipid species was observed for all types of cancer (Supplementary Fig. S2).

### Statistical evaluation of data

**Figure 7:**
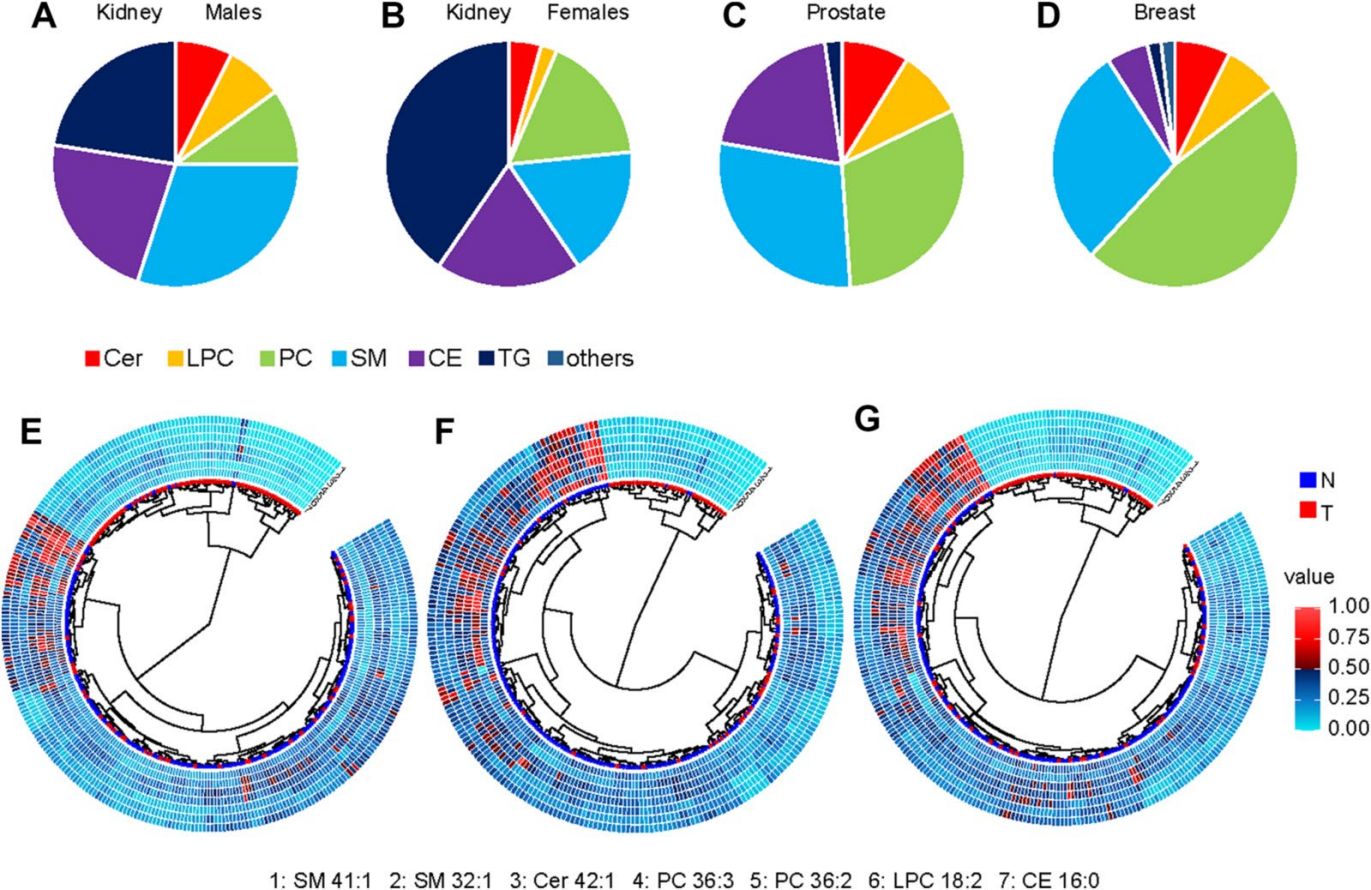
Distribution of lipid class percentage for individually lipid classes, which are significant according to the Bonferroni correction for the discovery phase: (**A**) kidney cancer males, (**B**) kidney cancer females, (**C**) prostate cancer males, and (**D**) breast cancer females. Dendrograms for 7 lipid species quantified in the discovery phase using UHPSFC/MS for both genders and training and validation sets for: (**E**) kidney cancer, (**F**) prostate cancer, and (**G**) breast cancer.

The different plasma lipidomic profiles depending on cancer type were investigated by evaluating statistically significant lipid species after Bonferroni correction, lipid species with a fold change of ± 20%, and VIP value > 1. The percentage of lipid species belonging to the lipid class fulfilling the defined criteria was calculated, as illustrated by the pie charts for different cancer types in Fig. 7A–D. Nonpolar lipid species, triacylglycerols and cholesterol esters, are of greater relevance in kidney cancer, while the influence of glycerophospholipids and sphingolipids appears to be dominant in breast and prostate cancer.

**Figure 8:**
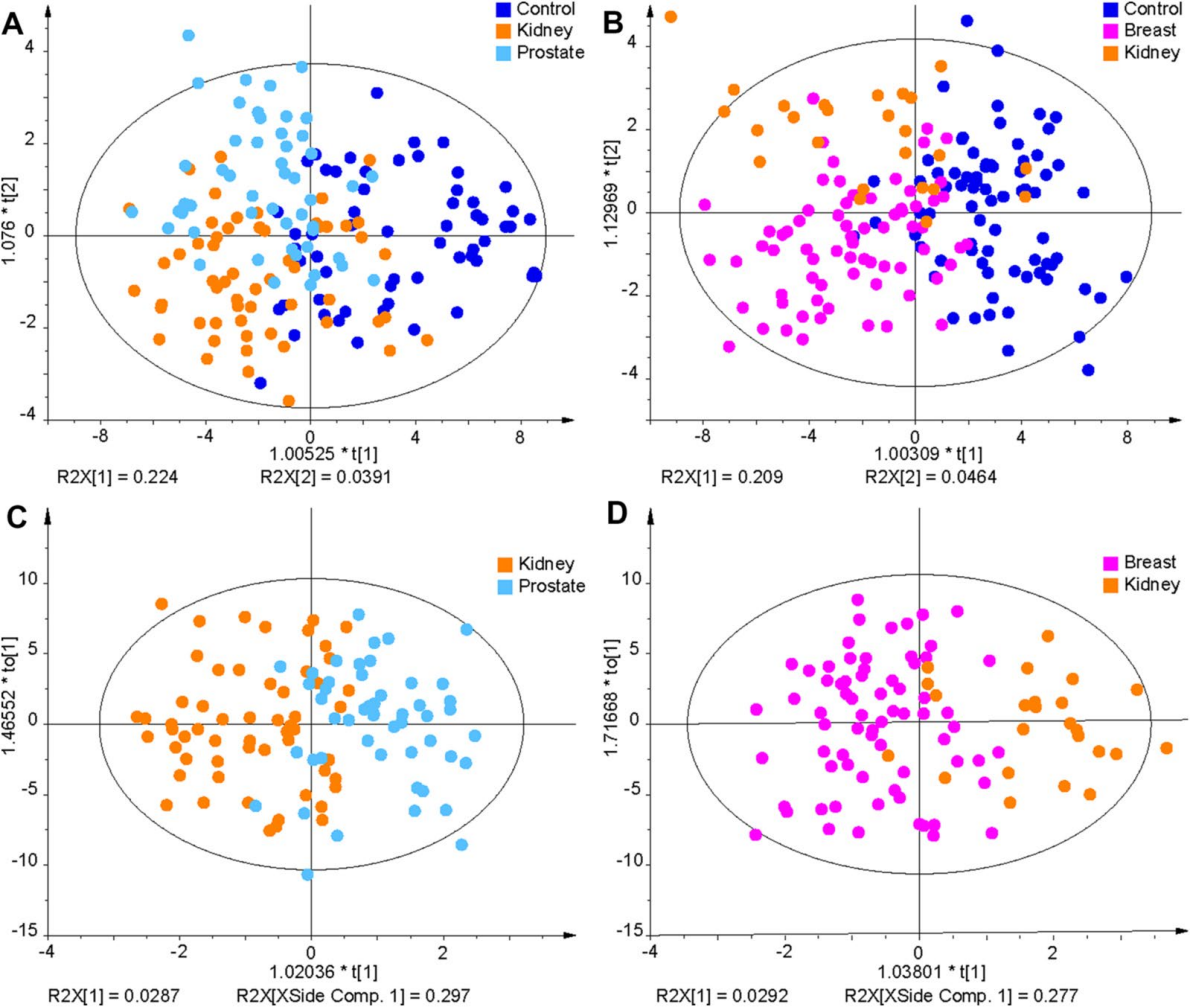
OPLS-DA models for the differentiation of the sample type like cancer type and control samples (**A**) males and (**B**) females using the concentrations of the 138 lipids determined in the discovery phase with UHPSFC/MS for the training set. OPLS-DA models for the differentiation of (**C**) prostate and kidney cancer samples for males and (**D**) breast and kidney cancer samples for females.

The most significant lipid species for all methods and data sets are downregulated in plasma samples of cancer patients, independent of the cancer type, as illustrated in Fig. 7E–G. MDA was used to investigate the differences between healthy control samples and different types of cancer types for all quantified lipid species, 91 lipid species for UHPSFC/MS and shotgun MS, 29, and finally, 7 most significant lipid species for all data sets. OPLS-DA models for samples obtained from healthy male controls and male patients suffering from prostate and kidney cancer as well as samples obtained from healthy female controls and female patients suffering from kidney and breast cancer are shown in Fig. 8A,B. The question was whether differentiation and prediction of cancer type and healthy control samples are possible using UHPSFC/MS. The specificity ranged from 67 to 97% with the average of 83%, sensitivity for kidney cancer from 49 to 74% with the average of 61%, sensitivity for prostate from 0 to 66% with the average of 43% and the accuracy from 57 to 74% with the average of 65% for the training and validation set and different numbers of lipids (138, 91, 29, and 7 lipid species) included to build the MDA models considering samples obtained from male donors (Fig. 8A, Supplementary Table S13). The specificity ranged from 80 to 96% with the mean of 90%, sensitivity for kidney cancer from 0 to 44% with the mean of 24%, sensitivity for breast cancer from 60 to 83% with the mean of 73% and the accuracy from 66 to 78% with the mean of 74% for the training and validation set and different numbers of lipids (138, 91, and 29 lipid species) included to build the MDA models considering samples obtained from female donors (Fig. 8B). It was not possible to perform the MDA model using 7 lipid species as variables due to the insufficient number of components for samples obtained from female donors.

The differentiation of the cancer type was also investigated by performing OPLS-DA models that classify kidney cancer versus prostate cancer for males (Fig. 8C) and kidney cancer versus breast cancer for females (Fig. 8D and Supplementary Table S14). OPLS-DA models were evaluated using 138 and 91 lipids as variables for males and 138, 91, and 29 for females using UHPSFC/MS data, since for the lower number of lipids a lack of components was observed. The sensitivity for prostate cancer was 71–88% with the mean of 81% and for kidney cancer 57–82% with the mean of 72% for the training and validation set and both UHPSFC/MS data sets considering male samples. The sensitivity for breast cancer ranged between 94 and 100% with the mean of 98%, the sensitivity for kidney cancer ranged between 14 and 86% with the mean of 57%, and the accuracy ranged between 80 and 97% with the mean of 88% for the training and validation set and both UHPSFC/MS data sets considering female samples.

## Discussion

Cancer screening as part of a regular health examination can allow early cancer detection and timely treatment, resulting in improved clinical outcomes. Circulating biomarker measurement as a minimally invasive and routinely used method seems to be one of the most attractive and convenient method for screening of high-risk individuals. Current approaches focus on the analysis of genetic mutations, ctDNA, or proteins for early cancer diagnosis in plasma or serum. To date, the clinical utility of lipidomic analysis for this purpose has not been clearly demonstrated.

In the present study, we performed quantitative lipidomics of human plasma samples collected from healthy controls and cancer patients by UHPSFC/MS. We paid special emphasis to the accurate molar quantitation of lipid species allowing the future interlaboratory comparison of the results if the same measurement protocol is applied.

MDA revealed the applicability of lipidomics as a diagnostic tool for all three cancer types studied. The performance of classification models in cancer prediction was characterized by high sensitivity, specificity, and accuracy. Nonpolar lipids, such as cholesteryl esters and triacylglycerols, are more important for kidney cancer, while differences in sphingolipid and glycerophospholipid profiles are more pronounced in breast and prostate cancers. The reduction in the number of quantified lipid species used for MDA showed only a slight loss in sensitivity, specificity, and accuracy. Nevertheless, the decrease in method complexity compared to the overall lipidomic profiling could facilitate potential clinical use.

Our results are consistent with previous reports on plasma or serum alterations in patients with different types of cancer^19^, including breast cancer^28–30^, pancreatic cancer^23,31^, kidney cancer^32^, lung cancer^33,34^, and prostate cancer^35,36^. We observed downregulation of multiple plasma lipid species in patients compared to healthy volunteers. Previous reports also showed the association of hypolipidemia with some malignancies^37^, but the peripheral blood lipidome may also be affected by other factors^38^.

Based on previous literature^39,40^, we hypothesize that the observed alterations in lipid concentrations are the overall result of complex processes in the human body, including the accumulation of lipids in plasma to favor tumor growth. Malignant cell proliferation requires excess lipids to build membranes, organelles, and participate in signaling processes^41^. The statistical analysis showed that the following seven lipids downregulated in patients' plasma had the most significant effect on the differentiation between cases and controls: CE 16:0, Cer 42:1, LPC 18:2, PC 36:2, PC 36:3, SM 32:1, and SM 41:1. CE 16:0 may be a potential source of palmitic acid for cancer cells. Palmitic acid can be converted to phospholipids, sphingolipids, glycerolipids, and other fatty acids essential for cancer cell survival. Louie et al. showed that cancer cells and tumors robustly incorporate exogenous palmitic acid and remodel it into other oncogenic lipid species^42^. Sphingolipids play an essential role in signal transduction pathways that regulate cell growth/death, migration, and senescence. Cancer cells can dysregulate enzymes involved in the metabolism of sphingolipids, resulting in the suppression of apoptosis, e.g., through downregulation of pro-cell death sphingolipids, such as ceramides^43,44^. LPC act as bioactive proinflammatory signaling molecules^45^. LPC are also substrates for LPA synthesis, and subsequently, LPA can modulate the immunological response and promote tumor cell growth^46^. PC are major components of membranes necessary for cell proliferation and survival. The alteration in PC metabolism is a potential signature of tumor progression and could be a good target for therapy^47^. The molecular biology techniques or MS-based approaches with stable isotope-labeled metabolites in future biological studies could shed more light on the lipid metabolism in cancer. Current data based on the analysis of lipids in human plasma could not explain the complete biological mechanism of ongoing processes in cancer.

Some previous studies have reported dysregulation of lipids in several diseases^17,48–50^. Unfortunately, molar concentrations are often not provided for measured lipids, therefore, the correlation of their conclusions with other studies is difficult. In any case, the future prospective study should include a group of cases with other nonmalignant conditions to determine whether the lipidomic analysis has the clinical utility for their differentiation from not only from healthy controls, but also from other diseases. It will also be worth investigating the discrimination potential of seven lipids versus the broader lipidomic profiling. In the present study, the accuracy decrease for cancer patients versus healthy controls is relatively low, but the comparison with patients with other nonmalignant diseases has not yet been performed.

In conclusion, the present data indicate the potential of lipid profiling in cancer screening, at least for breast, kidney, and prostate cancers. The use of individual MDA models to distinguish healthy control samples and the single cancer type results in higher accuracy than MDA models that include multiple cancer types. The use of IS for each lipid class allows the quantitation of lipid species and the comparison of lipid concentrations between different laboratories. Subsequent prospective studies are necessary for seven lipid species identified as potential biomarkers for cancer screening.

## Methods

### Human samples

A retrospective study was performed on 481 human plasma samples. A total of 192 control samples and 289 cancer samples from patients with breast, kidney, or prostate cancer were collected. The criteria for healthy controls were that they did not have any type of cancer during the life time and age over 18 years. For cancer patients, the disease was histologically confirmed by needle biopsy or by examining the surgical resection specimen. Both cancer patients and healthy controls were of the same ethnicity (Caucasian), collected in the same place (University Hospital in Olomouc) and processed in the same way. No other exclusion criteria were applied. The clinical information for all patients and controls is summarized in Fig. 1 and Supplementary Tables S1 and S2. The sample set was divided into training (using to build OPLS-DA models) and validation (indicates the possible use for samples with unknown classification) sets. Each fourth sample was assigned to the validation set, to obtain a distribution of the 75% of samples belonging to the training set and 25% of the samples to the validation set. Patients had no treatment before blood collection. Human plasma was collected in 9 mL lithium-heparin collection tubes and then centrifuged. The supernatant was transferred, aliquoted, and stored at − 80 °C until further processing for lipidomic analysis.

### Ethics declaration

The study was approved by the ethical committee of the University Hospital Olomouc. All subjects signed an informed consent. All methods were carried out in line with Ethical Principles for Medical Research Involving Human Subjects (Declaration of Helsinki).

### Study phases

The lipidome of 481 plasma samples was measured by UHPSFC/MS in the discovery phase. To ensure that UHPSFC/MS results are reproducible, the same extracts were measured again several months later corresponding to the qualification phase. The sequence of sample measurements was randomized to exclude any measurement bias. The data set was independently processed and the results were compared to the discovery phase. Furthermore, the extracts were also measured with shotgun MS to exclude any bias caused by the employed method, independently processed, and compared with UHPSFC/MS results.

### Chemicals

Solvents for analysis, such as acetonitrile, 2-propanol, methanol (HPLC/MS grade), water (UHPLC/MS grade), and hexane, were purchased from Honeywell (Riedel-da Haën, CHROMASOLV™ LC–MS Ultra, Hamburg, Germany), distributed by Fisher Scientific (Waltham, Massachusetts, USA). Chloroform stabilized with 0.5–1% ethanol was purchased from Sigma-Aldrich (St. Louis, MO, USA) or Merck (Darmstadt, Germany), respectively. Ammonium acetate was purchased from Fisher Scientific. Deionized water for liquid–liquid extraction was obtained from a Milli-Q Reference Water Purification System (Molsheim, France). Carbon dioxide of 4.5 grade (99.995%) was purchased from Messer Group (Bad Soden, Germany). Non-endogenous lipids were used as internal standards (IS) for quantitative analysis, *i.e.*, MG 19:1/0:0/0:0, DG 12:1/0:0/12:1, and TG 19:1/19:1/19:1 from Nu-ChekPrep (Elysian, MN, USA); CE 16:0 D7, Cer d18:1/12:0, cholesterol D7, LPC 17:0/0:0, LPE 14:0/0:0, PC 14:0/14:0, PC 22:1/22:1, PE 14:0/14:0, PI 15:0/18:1 D7, SM d18:1/12:0, PS 14:0/14:0, PA 14:0/14:0, PG 14:0/14:0, LPG 14:0/0:0, HexCer d18:1/12:0, Hex2Cer d18:1/12:0, and SHexCer d18:1/12:0 from Avanti Polar Lipids (Alabaster, AL, USA). The concentrations of stock solutions of individual IS and the volumes needed to prepare the IS mixture are summarized in Supplementary Table S15.

### Lipidomic analysis

For the extraction of lipids, a modified Folch procedure was employed, which was previously validated^51^. The same sample extracts were analyzed with UHPSFC/MS and shotgun MS. Human serum (25 µL) and the mixture of IS (17.5 µL) were homogenized in 3 mL of chloroform/methanol (2:1, *v/v*) for 10 min in an ultrasonic bath (40 °C). When the samples reached ambient temperature, 600 µL of water was added, and the mixture was vortexed for 1 min. After 3 min of centrifugation (3000 rpm), the aqueous layer was removed, and the organic layer was evaporated under a gentle stream of nitrogen. The residue was dissolved in a mixture of 500 µL of chloroform/2-propanol (1:1, *v/v*), carefully vortexed and filtered (0.2 µm syringe filter). The extract was diluted 1:20 with the mixture of hexane/2-propanol/chloroform (7:1.5:1.5, *v/v/v*) for UHPSFC/MS analysis and 1:8 with chloroform/methanol/2-propanol (1:2:4, *v/v/v*) mixture containing 7.5 mM of ammonium acetate and 1% of acetic acid for shotgun MS analysis.

UHPSFC/MS measurements were carried out on an Acquity Ultra Performance Convergence Chromatography (UPC2) system hyphenated to the hybrid quadrupole traveling wave ion mobility time-of-flight mass spectrometer Synapt G2-Si from Waters using the commercial interface kit (Waters, Milford, MA, USA). Chromatographic settings were used with minor improvements from the previously published method^51,52^. UHPSFC analyses were measured on the Viridis BEH column (100 × 3 mm, 1.7 µm) using a linear gradient with supercritical CO_2_ and as a modifier, MeOH with 30 mM ammonium acetate and 1% water: 0 min—1% modifier, 5 min—51% modifier, 6.5 min—51% modifier, 6.8 min—1% modifier. The total run time including the equilibration was 7.5 min. The automatic back-pressure regulator was set to 1800 psi, the column temperature to 60 °C, the flow to 1.9 mL/min, and the injection volume was 1 µL. The injection needle was washed after each injection with hexane/2-propanol/water (2:2:1, v/v/v). The make-up solvent was MeOH with 30 mM ammonium acetate and 1% water with the flow rate of 0.25 mL/min. The following parameters were set for MS measurements: the positive ion electrospray ionization (ESI) in the sensitivity mode, the mass range of m/z 150–1200, the capillary voltage of 3 kV, the sampling cone of 20 V, the source offset of 90 V, the source temperature of 150 °C, the desolvation temperature of 500 °C, the cone gas flow of 50 L/h, the desolvation gas flow of 1000 L/h, and the nebulizer gas flow of 4 bar. The analysis was done in the continuum mode with a scan time of 0.1 s and the lock mass scanning. Leucine enkephalin peptide was used as a lock mass with the scan time of 0.1 s and the interval of 30 s interval. The lock mass was scanned but the mass correction was not automatically applied. All samples were measured in duplicates. Noise reduction was performed on the raw files using the Waters compression tool. Data files were lock mass corrected and converted into centroid data using the exact mass measure tool from Waters. The MarkerLynx software from Waters was used for data preprocessing. Further data processing was done by LipidQuant 1.0 software^53^.

Shotgun experiments were performed on a 6500 QTRAP quadrupole linear ion trap mass spectrometer (Sciex, Concord, ON, Canada) equipped with an ESI probe using the characteristic precursor ion (PIS) and neutral loss (NL) scan events^54^. Raw data files were processed with the Sciex LipidView Software from Sciex in order to obtain a summary table of *m/z *versus intensity for each scan mode (NL and PIS) of all samples. Raw data were prefiltered by applying the following settings in the positive ion mode, a tolerance mass window of 0.5 Da, a minimum intensity threshold of 0.1%, and a minimum signal-to-noise ratio of 3 after smoothing. The summary tables of *m/z *versus intensity for all samples were exported as txt files and further processed by the LipidQuant 1.0 software.

### Data processing

LipidQuant 1.0 is a Microsoft Excel based script used for the automated data processing of txt files^53^ including *m/z* values versus intensities for all samples. The experimental *m/z* values were compared with the theoretical *m/z* values from the embedded database for lipid identification, depending on the retention time window or scan type defining the lipid class. The lipid quantitation was performed by calculating the ratio of the intensities of the target lipid and the internal standard and multiplying with the known concentration of the internal standard. Isotopic correction type II^55^ was automatically applied and a summary table containing lipid concentrations in all samples was generated. Zero filling for missing values was applied by setting the number for 80% of the minimum measured concentration for a given lipid species for all samples. If the concentration was not determined for more than 25% of the samples, then the lipid species was excluded from the data set. The data set was divided into training and validation set by assigning each fourth sample to the validation set. Clinical information for the samples, like gender and pathological state, was revealed, and samples were assigned. Final tables containing the lipid concentrations for all samples and fulfilling all defined criteria were used for MDA and other statistical tools.

### Statistical analysis

MDA was performed with SIMCA software, version 13.0 (Umetrics, Sweden). The lipid species were defined as variables, and the samples as observations. The data set was preprocessed by applying logarithmic transformation, pareto scaling, and centering. Data preprocessing should facilitate the normal distribution of lipid concentrations and that low abundant lipid species contribute similarly to the MDA as high abundant lipid species. PCA was performed to evaluate for outliers, estimate measurement quality by checking the clustering of QC samples, and evaluate the clustering of sample groups depending on the pathological state. OPLS-DA is a statistical tool for visualizing differences between sample groups of known classification. OPLS-DA was built using the training set and then used for the sample prediction of the validation set. For both PCA and OPLS-DA, the score scatter plots for the first two components are visualized, although more components may contribute to the model. The number of components for PCA and OPLS-DA models was determined by selecting the option autofit in the SIMCA software, where only components are considered of significance according to cross-validation rules. The cross-validation is automatically applied following Eastment et al*.* for PCA^56^ and Martens et al*.* for OPLS-DA^57^. The data set is divided into seven groups, omitting one group, building the model and predicting the excluded group. This is repeated for each group, and the results of predictions reveal the number of significant components, which is provided in Supplementary Table S4. OPLS-DA revealed differences in lipidome by using gender as a classifier. As a consequence, the data sets for females and males were treated separately for investigation of the prediction performance.

Microsoft Excel was used for the calculation of average lipid concentrations obtained for all sample groups, fold change, *T*-value, and *p*-value. For the calculation of *p*-value, a two-sided two-sample *T*-test assumed unequal variances (Welch test) for the samples obtained from healthy controls and patients with kidney, breast, or prostate cancer. *p*-values < 0.05 were considered as significant, but *p*-values were further evaluated according to the Bonferroni correction. All statistical parameters for all lipids are summarized in Supplementary Tables S3 and S6 below the lipid concentrations measured in individual samples and Supplementary Table S10. Another parameter indicating some relevance to differentiate samples from healthy controls and cancer patients, is the variable of importance (VIP) value obtained for each OPLS-DA plot. The most regulated and statistically significant lipid species with a fold change ± 20%, a *p*-value < 0.05, and a VIP value > 1 for all methods and phases are summarized in Supplementary Table S10. Box plots were used to better visualize lipid species concentrations depending on the health state. The box plots were constructed in R free software environment (https://www.r-project.org) using readxl and ggplot2 packages. In each box plot, the median was presented by a horizontal line, the box represented the first and the 3rd quartile values, and whiskers stood for 1.5*IQR from the median, and each measurement was plotted as a jittered point value. The receiver operating characteristics curves were generated by using the packages readxl and AUC in R. The dendrograms were also constructed in R^58^. For the circular dendrograms, the Euclidean distances were calculated, and then the upgma function from the phangorn library was used for clustering (the Ward agglomeration method was selected). Circular dendrograms were generated and surrounded by the heatmap (ggtree and gheatmap functions – ggtree library). For the presentation of the heatmap, all concentrations were min–max scaled.

## Supplementary Information

Supplementary Tables.

Supplementary Figures.

## Data Availability

All data relevant for the conclusions presented conclusions are provided in the manuscript or in the supplementary tables. Raw files of all measurements can be provided on request from the corresponding author.
