# Supplementary material for: Plasma lipidomic profiles of kidney, breast and prostate cancer patients differ from healthy controls
Source: Sci Rep. 2021 Oct 13;11:20322. doi: 10.1038/s41598-021-99586-1 (PMC8514434; doi:10.1038/s41598-021-99586-1)
Supplement: Supplementary file 2 — Supplementary Figures. [file 41598_2021_99586_MOESM2_ESM.pdf]

SUPPLEMENTARY INFORMATION

Plasma lipidomic profiles of kidney, breast, and prostate cancer patients differ from healthy controls

Denise Wolrab[1], Robert Jirásko[1], Ondřej Peterka[1], Jakub Idkowiak[1], Michaela Chocholoušková[1], Zuzana Vaňková[1], Karel Hořejší,[1] Ivana Brabcová,[1] David Vrána,[2,3] Hana Študentová[2], Bohuslav Melichar[2], Michal Holčapek[1,*]

[1] *University of Pardubice, Faculty of Chemical Technology, Department of Analytical Chemistry, Studentská 573, 532 10 Pardubice, Czech Republic*

[2] *Department of Oncology, Faculty of Medicine and Dentistry, Palacký University and University Hospital, I.P. Pavlova 6, 775 20 Olomouc, Czech Republic*

[3] *Current address: Hospital Nový Jičín, Comprehensive Cancer Center Nový Jičín, Czech Republic*

* Corresponding Author: Michal Holčapek

E-mail: Michal.Holcapek@upce.cz

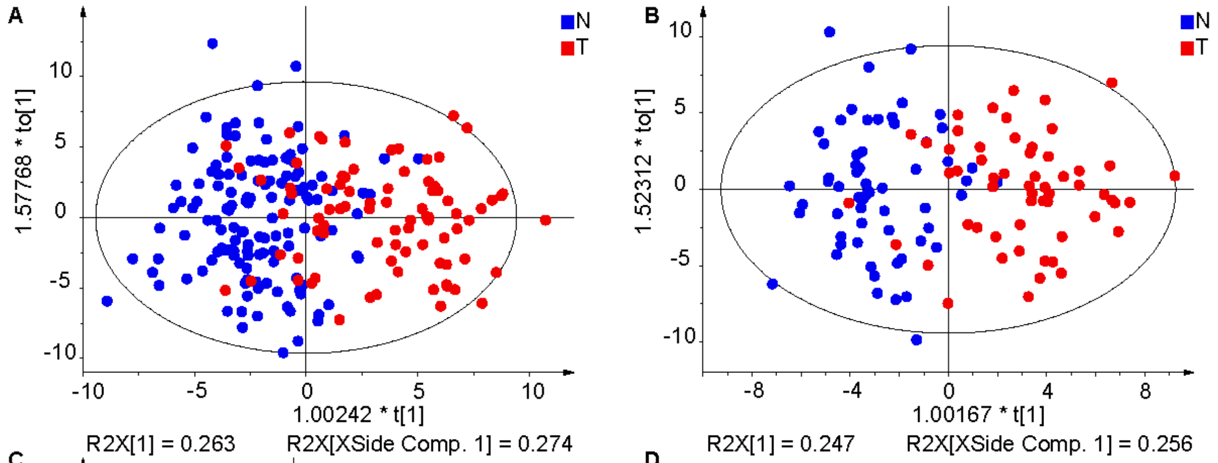

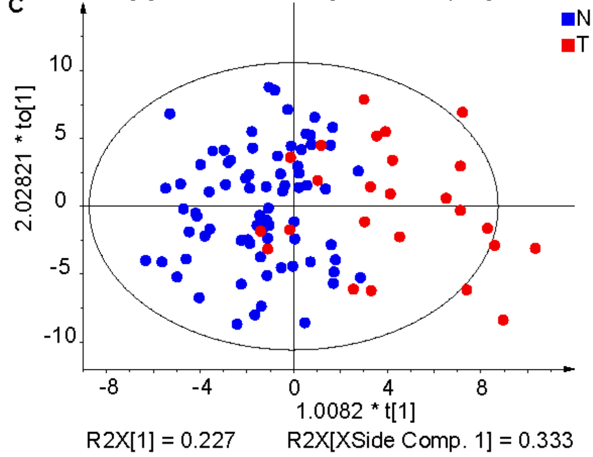

D

	Training set		
	M	F	both
Specificity	93.3	98.7	94.1
Sensitivity	86.0	72.0	72.0
Accuracy	89.7	92.0	85.7
	Validation set		
Specificity	93.3	98.7	94.1
Sensitivity	86.0	72.0	72.0
Accuracy	89.7	92.0	85.7

Supplementary Fig. 1. Comparison of parameters of OPLS-DA statistical models prepared for: **a** both genders, **b** only males, **c** only females, and **d** specificity, sensitivity, and accuracy for all models.

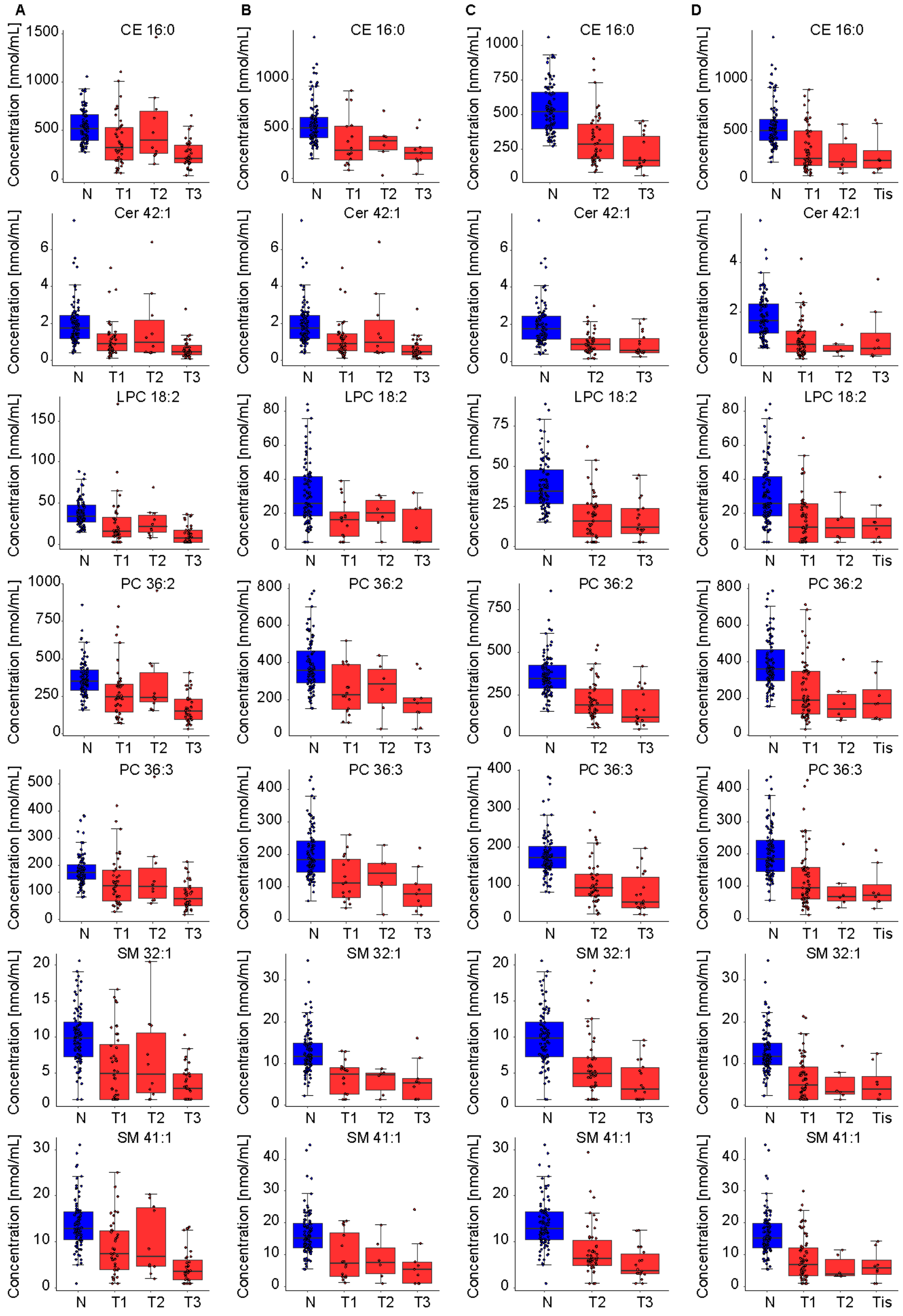

Supplementary Fig. 2. Box plots for seven most dysregulated lipid species shown separately for: **a** kidney cancer males, **b** kidney cancer females, **c** breast cancer, and **d** prostate cancer.
